# Immune cell-mediated features of atherosclerosis

**DOI:** 10.3389/fcvm.2024.1450737

**Published:** 2024-08-21

**Authors:** Tingting Liu, Yanjun Chen, Lianjie Hou, Yulu Yu, Dan Ma, Ting Jiang, Guojun Zhao

**Affiliations:** ^1^ Affiliated Qingyuan Hospital, Guangzhou Medical University, Qingyuan People’s Hospital, Qingyuan, Guangdong, China; ^2^ Department of Pathology, Southwest Hospital, Third Military Medical University, Chongqing, China; ^3^ School of Pharmacy, Zunyi Medical University, Zunyi, Guizhou, China

**Keywords:** immune cells, atherosclerosis, targeted therapy, cardiovascular diseases, inflammation

## Abstract

Atherosclerosis is a chronic inflammatory disease characterized by innate and adaptive immune responses, which seriously threatens human life and health. It is a primary cause of coronary heart disease, myocardial infarction, and peripheral vascular disease. Research has demonstrated that immune cells are fundamental to the development of atherosclerosis and chronic inflammation. Therefore, it is anticipated that immunotherapy targeting immune cells will be a novel technique in the management of atherosclerosis. This article reviews the growth of research on the regulatory role of immune cells in atherosclerosis and targeted therapy approaches. The purpose is to offer new therapeutic approaches for the control and treatment of cardiovascular illnesses caused by atherosclerosis.

## Introduction

Global mortality and disability statistics are dominated by cardiovascular diseases, which include peripheral artery disease, myocardial infarction, stroke, and coronary artery disease (1). The pathology known as atherosclerosis (AS), which is common to many cardiovascular diseases, is a chronic inflammatory condition caused by the buildup of immune cell-rich, lipid-filled plaques in large and medium-sized arteries. In preclinical models, the immune system and inflammation have a well-established causative role in AS (2). Recently, the Canakinumab Anti-inflammatory Thrombotic Outcome Study (CANTOS) provides the first substantial evidence that it is feasible to treat the inflammatory component of atherosclerotic thrombosis by examining the effect of IL-1β inhibition on immune regulation (3). However, in this trial, systemic immune suppression led to a greater frequency of infections in the treatment group compared to the placebo group, increasing the risk of major adverse events and even death.

Atherosclerotic cardiovascular disease patients' systemic immunological changes have been the focus of most current studies (4). Moreover, little is known about the cellular composition of human atherosclerotic plaques, where clinical events are caused by the plaque rupture. The significance of immune cell plasticity in AS has been extensively researched using animal models, where immune cells acquire pro-atherogenic and anti-atherogenic functions in a range of differentiation phases that determine the destiny of an atherosclerotic lesion (5). Nevertheless, the identification of the dynamic changes and interactions between diverse immune cell subsets in the human atherosclerotic plaque has been limited to low-dimensional methods that fail to capture the coordinated activity of heterogeneous cells in both healthy and diseased conditions.

The first description of immune cells in the arterial wall using immunohistochemical methods since the early 1980s, and it provided important information on immune cells invading atherosclerotic lesions (6). Following this, the development of flow cytometry has made it easier to identify particular immune cell phenotypes and rare subpopulations (7). The single-cell technologies that have advanced dramatically over the last ten years include single- cell mass cytometry (cytometry by time of flight; CyTOF) (8), single- cell RNA sequencing (scRNA-seq) (9) and cellular indexing of transcriptomes and epitopes by sequencing (CITE-seq) (10). Depuydt et al. (11), Fernandez et al. (12), and Vallejo et al. (13) used scRNA-seq to analyse immune cells in human atherosclerotic lesions and found three major immune cell subpopulations in macrophages: resident-like macrophages, inflammatory macrophages, and TREM-2^hi^ macrophages; three major immune cell subpopulations in dendritic cells: cDC1, cDC2, and Mature DC; four major immune cell subpopulations in T cells: Naïve T cells, Cytotoxic T cells, Regulatory T cells, and Memory T cells; and two major immune cell subpopulations in B cells: Naïve B cells, and Memory B cells. The development of new technologies and the improvement of current techniques have led to an increase in the accuracy of research on immune cell subpopulations that penetrate atherosclerotic lesions. This will allow for the development of immune cell-targeted medicines to more precisely regulate AS. In this review, we will summarize the regulatory mechanisms of different immune cells in the pathological process of AS and targeted therapies against the immune cell and molecular mechanisms of AS, laying the foundation for finding more efficient, targeted and safer immune cell-based therapy for AS.

## Pathophysiology of atherosclerosis

**Figure 1:**
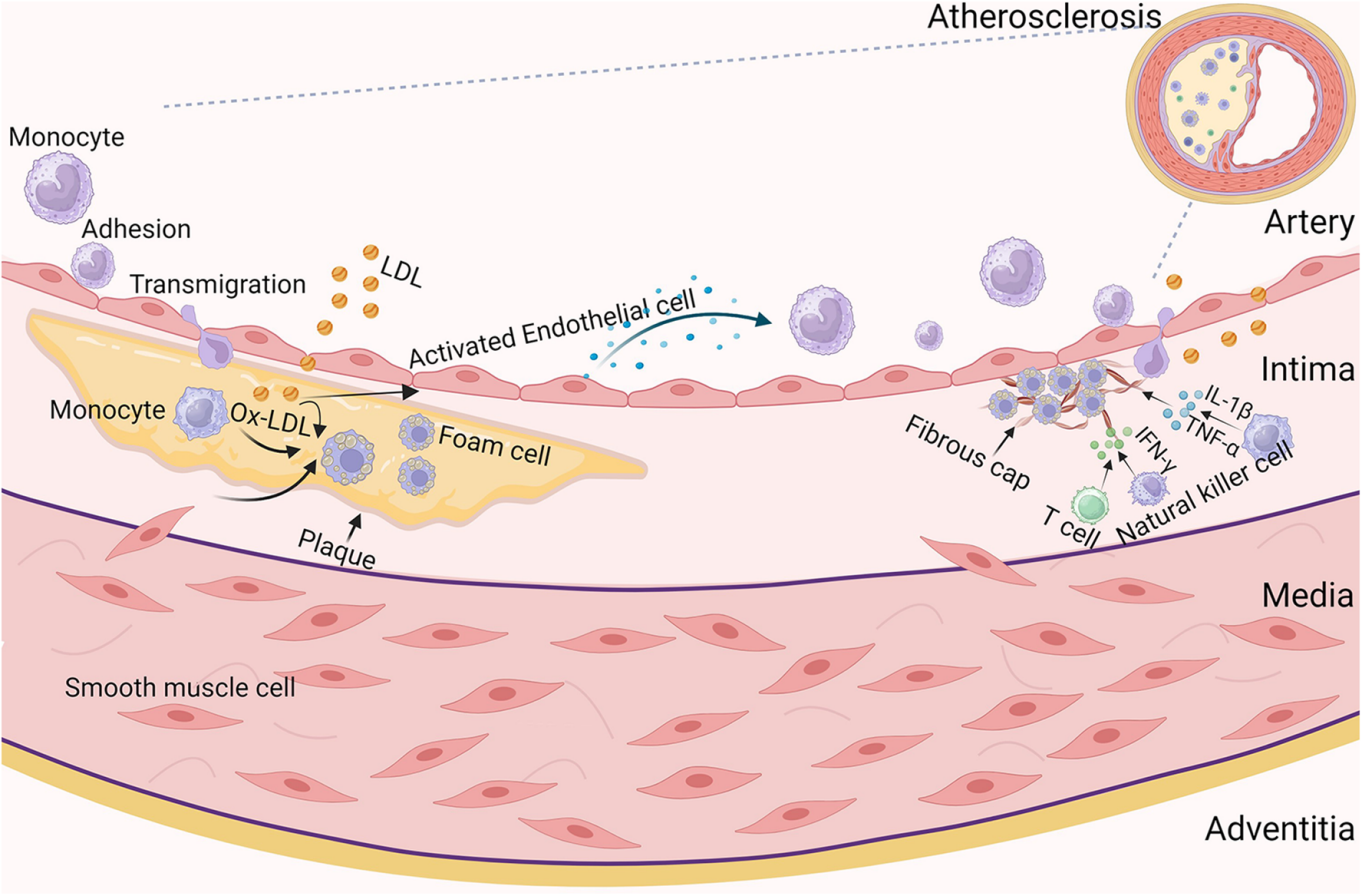
Pathogenetic processes of atherosclerosis. Atherosclerosis is a progressive, multifactorial inflammatory disease. It involves endothelial activation, monocyte recruitment, macrophages, vascular endothelial cell activation and proliferation, antigen presentation to T cells, and ultimately increased secretion of pro-inflammatory cytokines, inflammation and necrosis. In the early stages of atherosclerosis, vascular endothelial cell injury leads to infiltration of low-density lipoprotein (LDL) into the intima, where it undergoes oxidative modification, activates vascular endothelial cells to release chemokines, promotes monocyte recruitment, adherence, penetration of the intima and differentiation into macrophages. During lesion growth, vascular smooth muscle cells in the media move from to the intima. Macrophages and smooth muscle cells in the intima capture oxidised low-density lipoproteins, which then form foam cells retained in the plaque. To stabilise the plaque, the necrotic core is covered by fibres, thus forming a fibrous cap. During this period, T cells and natural killer cells are activated and produce mediators such as IFN-γ, and macrophages produce IL-1β and TNF-α, further exacerbating the inflammatory response and leading to atherosclerosis.

AS begins with endothelial cell (EC) damage, followed by lipid accumulation, fibrosis and calcification, which in turn leads to lumen narrowing and a range of cardiovascular complications (14). Early on in the development of AS, low-density lipoprotein (LDL) infiltrates the intima, where it undergoes oxidative modification, activates vascular ECs to release chemokines, and promotes monocyte recruitment to penetrate the intima and differentiate into macrophages, which evolve into foam cells after taking up large amounts of oxidized LDL (Ox-LDL). Vascular smooth muscle cells (VSMCs) in the media change from a contractile state to a proliferative state during lesion growth and migrate into the intima to capture Ox-LDL, forming foam cells retained in the plaque (15). During fibrous plaque formation, a necrotic core that is rich in lipids and devoid of cells forms. Fibers cover the necrotic core to produce a fibrous cap that stabilizes the plaque (16). In addition, some proinflammatory cytokines, such as interferon gamma (IFN-γ), may prevent vascular ECs from producing collagen. Furthermore, inflammatory mediators like tumour necrosis factor alpha (TNF-α) and interleukin-1β (IL-1β) that are often present in atherosclerotic plaques and may trigger the production of matrix metalloproteinase (MMP) in VSMCs (17) (Figure 1). This inflammatory phase is typically observed in the plaque's cap and shoulders, rather than systemic inflammation (18). All of these findings point to the possibility that when inflammation is prevalent, the fibrous cap's capacity to remain robust is compromised, rendering it unstable and more likely to burst when exposed to hemodynamic stresses (19). Plaque rupture is an injury to the plaque where there is a real defect or gap in the fibrous cap that divides the lipid-rich atherosclerotic core from the flowing blood, thus exposing the thrombogenic core of the plaque (16). This further promotes platelet and thrombus aggregation, which increases the risk of vascular occlusion and leads to a reduction in coronary blood flow, thus increasing the risk of diseases such as ischaemic heart disease (heart failure) and angina pectoris (20). Furthermore, during this phase, interstitial collagen synthesis might be stimulated, which is important for the stabilization of platelet-rich thrombus development later on. When a blood clot forms that clogs or nearly clogs an artery, it can lead to a myocardial infarction or stroke (21). Blood clots dislodged from the arterial wall form emboli that circulate through the cardiovascular system. The emboli eventually become lodged in the distal arteries, blocking blood flow and perhaps causing infarction, organ failure, or localized ischemia (22).

## Cardiovascular immune landscape

Immune cells are mainly located in the adventitia. Dendritic cells (DCs) act as antigen-presenting cells and are involved in the activation of T cell-mediated adaptive immune responses. It has been found that dendritic cells at a steady state accumulate mainly at sites of blood turbulence (23), predicting that the distribution of dendritic cells may be related to hemodynamics. In vascular homeostasis, local macrophages in adult mouse arteries are maintained primarily by self-renewal and are not dependent on replenishment of the circulating monocyte supply. In the vasculature, macrophages are predominantly located in the outer membrane and play a regulatory role by recognizing antigens and presenting them to T cells (24). In addition, macrophages can play a pro-inflammatory or anti-inflammatory role by secreting different cytokines to remove necrotic tissue and promote injury repair (25). T cells, which play a crucial role in the adaptive immune response, develop and differentiate in the thymus before being carried by the humoral circulation to immunological organs and tissues throughout the body to carry out vital immune tasks. B cells are also a major component of the adaptive immune response and in addition to being involved in humoral immunity, B cells can also modulate cellular immunity by presenting antigens. Locally in arteries, T and B cells are predominantly located in the outer membrane (26). The researchers found that in wild-type and Apoe^−/−^ mice, the infiltration of arteriolar epithelial T and B cells increased with aging, and genes linked to B-cells, including Ighm, Ptpn6, and Lilrb3, have increased expression (27). This reveals that natural aging of the vasculature at homeostasis may be accompanied by alterations in the local immune cell microenvironment, thereby affecting local arterial T cell proliferation or recruitment and B cell phenotypic activation. In response to inflammatory or injurious stimuli, various immune cells exert different immunomodulatory effects and interact with each other to maintain vascular homeostasis.

## Immune modulation of atherosclerosis pathogenesis

The pathogenesis of atherosclerosis is a process of vascular damage caused by the interaction of cells in the blood vessel wall and blood cells with cytokines. Therefore, the response of the body's immune cells is the first step in the formation of AS plaques and is one of the main causes of atheromatous plaque instability. This includes humoral and cellular immune responses that are carried out by common dendritic cells, T cells, B cells, monocytes/macrophages, and other immune cells. Several clinical trials have also shown that the level of immune cells in the body is related to atherosclerotic plaques (12). Therefore, studying the regulatory characteristics of immune cells in AS is expected to prevent and treat AS from the perspective of immunotherapy.

### Dendritic cells

DCs are expert antigen-presenting cells (APCs) that facilitate the initiation and advancement of AS. Although they can be seen in a healthy aorta, as the illness worsens, their quantity increases. Rather than being equally dispersed throughout the vascular tissues, DC-like cells were discovered to be mostly collected in aortic wall regions that are prone to AS (28).

DCs work in subpopulations that exert different atherosclerotic effects by expressing different surface markers (Table 1). Two phenotypically different groups of DCs were identified in normal intima: CD11c^+^CD11b^−^CD103^+^and CD11c^+^CD11b^+^CD103^−^ DCs (29). It was discovered that the atherosclerotic plaque was was primarily composed of this CD11b^+^ typical DCs subgroup. In Apoe^−/−^ mice, CD11b^+^ DCs promote AS by triggering inflammation in receptor ECs via exosomal membrane-bound TNF-α or interacting with circulating Natural Killer T Cells (NKT) and Tregs (30). Fms-like tyrosine kinase 3 (Flt3l) signaling is essential for the growth and survival of vascular CD103^+^ DCs. A high-fat diet was given to Flt3l/low-density lipoprotein receptor-deficient (Flt3^−/−^Ldlr^−/−^) mice, and loss of this DCs subset resulted in decreased levels of anti-inflammatory IL-10, reduced aortic Tregs, and increased production of proinflammatory cytokines like interferon IFN-γ and tumor necrosis factor TNF-α, accelerating the development of AS (31). Furthermore, by promoting Treg development and recruitment through the CCL22-TGF-β pathway, or retinoic acid, vascular CD103^+^ DCs can also have anti-atherosclerotic effects in mice (32). Plasmacytoid DCs (pDCs) are the third subset of DCs that have been linked to the onset of AS. This fraction of pDCs secretes high levels of type 1 interferons, IFN-α and IFN-β, which are potent proinflammatory cytokines that promote the development of AS in patients with CAD (33). Moreover, specific deletion of pDCs of MHCII molecules in Ldlr^−/−^ mice results in decreased activation of CD4^+^T cells, lower production of the inflammatory cytokine IFN-γ by T cells, and lower migration of T cells to the lesion site (34). Additionally, pDCs within the plaques can stimulate myeloid dendritic cells to produce TLR4, TNF-α, and IL-12, which enhances the function of CD8^+^ T cells within human plaques, thereby promoting atherosclerotic effects (35). A study demonstrated that CCL17^+^ DCs, a different subgroup of DCs, were present in atherosclerotic lesions close to the aortic root but not in the healthy vascular wall. Demonstration in mice that CCL17-deficient atherosclerotic mice cause Treg amplification in plaques and a reduction in T-cell content in plaques, which reduces AS progression (36). Additionally, CCL17^+^ DCs in mice showed higher levels of the co-stimulatory markers CD40, CD80 and CD86 (37), suggesting that they may be able to promote AS through *in situ* activation of CD4^+^ T cells. In conclusion, among the different subtypes of DCs, CD11b^+^ DCs, pDCs and CCL17^+^ DCs exerted pro-atherosclerotic effects. CD103^+^ DCs exerted anti-atherosclerotic effects.

**Table 1:** Distinct vascular DC subsets in atherogenesis.

	CD11b^+^ DCs	CD103^+^ DCs	pDCs	CCL17^+^ DCs
Major functional molecules	TNF-α	IL-10, IFN-γ and TNF-α	IFN-α, IFN-β, IFN-γ, TLR4 and IL-12	CD40, CD80 and CD86
Immune reactions	Initiation of inflammation of the receptor endothelial cells; Interaction with circulating natural killer T cells (NKT) and Tregs	Tregs activation; Promotion of IL-10 secretion; Inhibition of IFN-γ, TNF-α secretion	CD4^+^T cell, CD8^+^T cell activation; Promotion of inflammation	CD4^+^T cell activation; Inhibition of Tregs amplification
Possible pathways	/	TGF-β/retinoic acid or CCL22	/	/
Roles in atherosclerosis	Pro-atherogenic	Anti-atherogenic	Pro-atherogenic	Pro-atherogenic

Targeting DCs presents a potential avenue for atherosclerosis vaccination. A potential autoantigen in AS, Ox-LDL or ApoB, was loaded onto GM-DCs (bone marrow-derived DCs grown with GM-CSF) with an immature phenotype, and these GM-DCs were then adoptively transferred into Ldlr^−/−^ mice. These therapies reduce infiltration of lesions by CD4^+^ T cells, increase the production of Tregs, and reduce the size of atherosclerotic plaques in mice (38, 39). In contrast to immature DCs, Apoe^−/−^ mice received mature GM-DCs loaded with the modified autoantigen MDA-LDL (malondialdehyde-modified LDL), with aggravation of atherosclerotic plaques but no induction of Tregs (40). These pre-clinical studies suggest that DCs-based vaccination may be an effective method for triggering protective Tregs response in atherosclerosis treatment. To achieve this goal, new approaches are needed to identify the antigenic specificity of reactive T cells in atherosclerotic lesions in order to design effective immunosuppressive vaccines.

Despite immunotherapy targeting DCs as an immune vaccine has been demonstrated to reduce AS. However, *in vitro* cultured GM-DCs are a heterozygous population of DCs and macrophages with much less immunostimulatory capacity than true DCs (41). Therefore, immune-targeted therapies that directly target DCs *in vivo* may have more potential in the treatment of AS. For instance, the nanocarriers were used to transport the anti-inflammatory agent 1,25-Dihydroxyvitamin D3 (aVD) and the apolipoprotein B-100-derived antigenic peptide P210 to selectively target and modulate DCs. The addition of P210 peptide decreased vascular lesion area, macrophage content, and vascular stiffness (42). In addition, regulatory effects can be exerted by affecting the biological functions of specific cells. Under high-fat dietary conditions, disruption of autophagy by deletion of Atg16l1 differentially affected the biology and function of DCs subpopulations in Ldlr^−/−^ mice. Deletion of Atg16l1 in a subpopulation of CD11b^+^ DCs expands CD4^+^ regulatory T cells, restricts the growth of type 1T helper cells, and limits the development of atherosclerosis. However, no such consequences were observed when Atg16l1 was specifically deleted in CD103^+^ and conventional CD8*α*^+^ DCs (43).

All of these findings suggest that targeting the right cells or molecules with DC-based immunization may be a more effective way to treat AS. Therefore, in order to create immunosuppressive vaccines that work, novel methods for identifying atherogenic autoantigens or compromised immune pathways in atherosclerotic lesions are required.

### Monocytes and macrophages

Monocytes are blood cells with a brief lifespan that are produced in the bone marrow. Once in circulation, they can develop into long-lived macrophages and produce inflammatory cytokines. Different macrophage subtypes all have different roles in plaque formation. It starts to form when macrophages that dwell in the intima soak up extra lipids and produce foam cells. Continuous blood circulation and monocyte inflow, which promote plaque macrophage formation, are necessary for further plaque advancement (44).

One characteristic that sets macrophage apart is their plasticity, which enables them to respond specifically to stimuli in the local microenvironment to either resolve or perpetuate inflammation (45). According to cytokine-induced *in vitro* settings, macrophage phenotypes are classified into two categories (M1 and M2) in the conventional, simplistic categorization. M1 macrophages are pro-inflammatory macrophages that are crucial for host defense and the release of pro-inflammatory cytokines. M2 macrophages have been reported to help with tissue regeneration and reduce inflammation (46). In the setting of plaques, macrophages adhering to both the classically activated and alternatively activated subsets are seen in both human and mouse lesions (47). Apoptosis of macrophages is triggered by persistent inflammation, which also causes a buildup of apoptotic cells and debris in the absence of efficient efferocytosis, promoting the development of a plaque necrotic core. In human lesions, M2 macrophage-representing cells are found in stable plaque locations, while macrophages expressing proinflammatory markers are found in rupture-prone, unstable regions (48). A growing body of research indicates that macrophage heterogeneity inside plaques is oversimplified by the M1/M2 categorization scheme and that macrophages are located on an activation continuum (49). There are other subtypes of macrophages, such as antiatherogenic Mhem macrophages and atherogenic Mox and M4 macrophages (50). According to recent research, the advanced plaques found in Ldlr^−/−^ mice are made up of a variety of macrophage populations, such as Mox macrophages, CD206^+^ M2-like macrophages, and CD86^high^ M1-like macrophages. While atherosclerosis involves the coexistence of macrophages in different active states, research has shown that pro-inflammatory macrophages (M1-like) are more frequently found in the plaque shoulder, which is the region most vulnerable to unstable plaque rupture (48).

**Figure 2:**
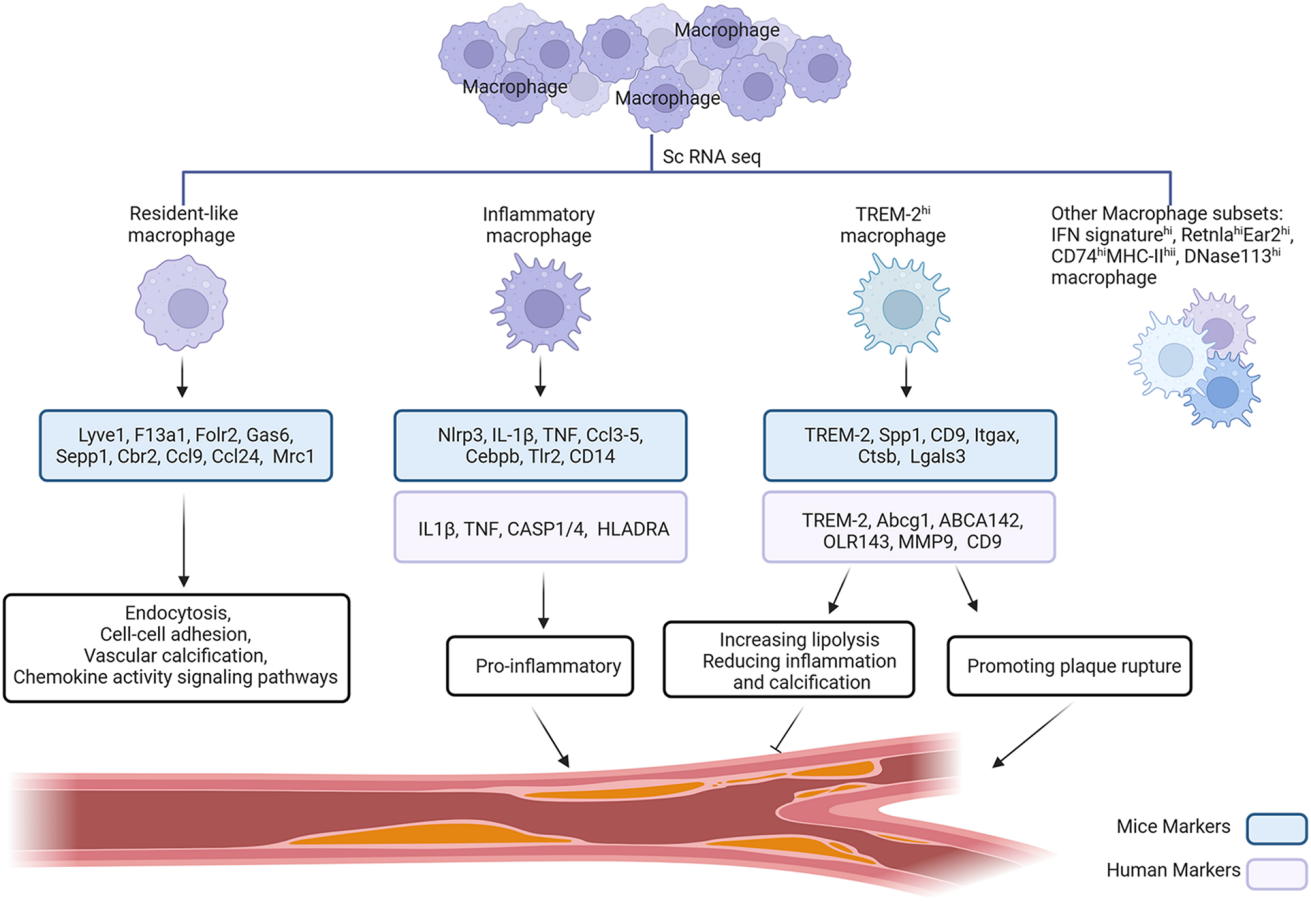
Identification of major mice and human macrophages in atherosclerosis by scRNA-Seq. ScRNA-Seq identified three major mice and human macrophage subpopulations. Resident-like macrophages, a subpopulation of macrophages expressing genes such as Lyve1 and F13a1, are primarily involved in endocytosis, cell-cell adhesion, vascular calcification and chemokine activity signalling pathways. Inflammatory macrophages produce a variety of pro-inflammatory molecules, including Nlrp3 and IL-1β, suggesting their inflammatory role in AS. TREM-2^hi^ macrophage markers include TREM-2, Spp1, and others. By reducing inflammation and calcification and enhancing lipolysis, it could have anti-atherosclerotic effects; nonetheless, AS and plaque rupture are linked to certain risk factors. In addition to the three major macrophage subpopulations mentioned above, there are other macrophage subpopulations such as IFN signature^hi^, Retnla^hi^Ear2^hi^ macrophages, etc.

Macrophages in atherosclerotic plaques were earlier classified as M1 or M2 type macrophages based on their phenotype regulated by microenvironmental changes such as lipid modifications and cytokines. Numerous studies have examined macrophages in mice atherosclerotic plaques using scRNA-seq, a novel technology that has been developed and applied in recent years. Relevant studies have shown that macrophages can be divided into at least three subpopulations: resident-like macrophages, inflammatory macrophages, and TREM-2^hi^ macrophages (51). The number of macrophage subpopulations in AS found in different studies varies due to the different experimental protocols employed. The studies by Kim et al. (52) and Lin et al. (53) emphasised or focused directly on macrophages. Thus, apart from the three subpopulations that symbolize the general inflammatory characteristics of atherosclerosis, these researchers discovered additional macrophage activation states in AS, including IFN signature^hi^, Retnla^hi^Ear2^hi^, CD74^hi^MHC-II^hi^, and DNase113^hi^ macrophages in the progression of plaques, as well as plaque regression HSP^hi^ and Ebf1^hi^Cd79a^hi^ macrophages (Figure 2).

Several single-cell studies have found resident-like macrophages in healthy mouse arteries and atherosclerotic vessels (51). The percentage of resident-like macrophages in advancing and regressed plaques was the same. The genes lymphatic vessel endothelial hyaluronan receptor 1 (Lyve1), factor XIII-a (F13a1), folate receptor β (Folr2), growth arrest-specific 6 (Gas6), selenoprotein P (Sepp1), carbonyl reductase 2 (Cbr2), carbonyl reductase 9 (Ccl9), and Mrc1 were expressed by this subgroup (53). This subpopulation is primarily engaged in endocytosis, cell-cell adhesion, vascular calcification, and chemokine activity signaling pathways, according to gene enrichment analysis (54). Modified lipoproteins in the atherosclerotic arterial wall stimulate resident macrophages and draw in monocyte-derived cells in atherosclerosis (55). When proinflammatory cytokines and modified lipoproteins are abundant, CCR2^+^ macrophages become attracted to and polarized toward a proinflammatory phenotype; these cells are referred to as inflammatory macrophages (56). Inflammatory macrophages mainly accumulate at atherosclerotic lesion sites. Numerous pro-inflammatory molecules, including nucleotide-binding structural domain and leucine-rich repetitive heat protein-containing 3 structural domains (Nlrp3), IL-1β, TNF, Ccl3-5, CCAAT enhancer-binding protein beta (Cebpb), Tlr2, and CD14, are produced by this subpopulation, indicating an inflammatory function in atherosclerosis (51). TREM-2^hi^ macrophages are a new macrophage subtype identified by scRNA-seq. These macrophages exclusively cluster in the plaque intima and not in the healthy aorta (51). Markers for this subpopulation of TREM2^hi^ macrophages include TREM-2, secreted phosphoprotein 1 (Spp1), CD9, Itgax, and galectin-3 (Lgals3) (53). The TREM-2^hi^ macrophage subtype may have an anti-atherosclerotic function in atherosclerotic plaques by increasing lipolysis (57) and reducing inflammation and calcification (58); nevertheless, AS and plaque rupture are also related with risk factors (59). Consequently, more research is needed to determine the precise function of this subgroup.

Similar findings were made by a number of investigations using scRNA-seq on human carotid atherosclerotic plaque cells, which revealed that distinct functional states were displayed by macrophage subpopulations in the plaques. A subset of macrophages was found to express marker genes TREM-2, ATP binding cassette subfamily G member 1(Abcg1), ABCA142, OLR143, matrix metalloproteinase 9 (MMP9), and CD9. These subpopulations' gene expression patterns resembled those of TREM-2^hi^ macrophages in mice atherosclerosis (11). The hallmark genes for inflammation, IL-1β, TNF, CASP1/4, and HLADRA, were expressed by certain subpopulations of macrophages. These subpopulations' gene expression patterns could resemble those of inflammatory macrophages in mice atherosclerosis (11). In conclusion, human macrophages exhibit distinct states in atherosclerotic plaques, which is in line with mice macrophages. Further research is required in the future to clarify the precise function of various macrophage subpopulations in atherosclerosis, which might lead to the discovery of novel targets to slow the advancement of AS.

Lipid modulation strategies and antiatherosclerotic biomarkers are measures of non-specific interventions that inhibit the role of macrophages in plaque development. Nevertheless, there are few treatments that target macrophages directly and precisely. At the moment, it is feasible to specifically alter macrophages thanks to innovative drug delivery methods. A novel conjugation of hyaluronan (HA) and atorvastatin (ATV) was created, with the hydrophobic statin ATV serving as the nanoparticle's core (HA-ATV-NP). The anti-inflammatory effects of HA-ATV-NPs on macrophages were considerably greater *in vitro* than those of ATV alone. Additionally, in an Apoe^−/−^ mouse model of atherosclerosis, HA-ATV-NP dramatically decreased the inflammatory response of advanced atherosclerotic plaques (60). Additionally, rapamycin-loaded poly (lactic-co-ethanolic acid) copolymer (PLGA) nanoparticles (RAPNPs) were coated with macrophage membranes (MM) to create biomimetic nanoparticles (MM/RAPNPs), which were based on macrophage “homing” into atherosclerotic lesions and cell membrane-covered nanotechnology. *in vitro*, these nanoparticles target active macrophages and efficiently prevent macrophage phagocytosis. Atherosclerotic lesions are an efficient target for MM-coated nanoparticles, which can aggregate there and slow the progression of AS in Apoe^−/−^ mice (61). On the basis of targeting macrophages for their role in modulating the atherosclerotic process, dual therapies with similar nanoparticle-targeted drug delivery could improve efficacy. For instance, Kim and associates created cargo-switching nanoparticles that have the ability to scavenge intracellular cholesterol and simultaneously release simvastatin from the phospholipid core-shell nanoparticles of methyl-β-cyclodextrin (62). Using a cargo-switching approach, Apoe^−/−^ mice were able to achieve synergistic anti-atherogenic efficaciousness by depleting pre-existing cholesterol in plaques and delivering simvastatin locally to lesional macrophages. Another method of managing macrophage function is to affect their polarization towards an anti-inflammatory phenotype, namely M2 macrophages. Any element that modifies M2 polarization signals might be a potential target. Nevertheless, research conducted thus far has only been preclinical, maybe as a result of the intricate phenotypic and functional variability of lesional macrophages.

### T cells

**Figure 3:**
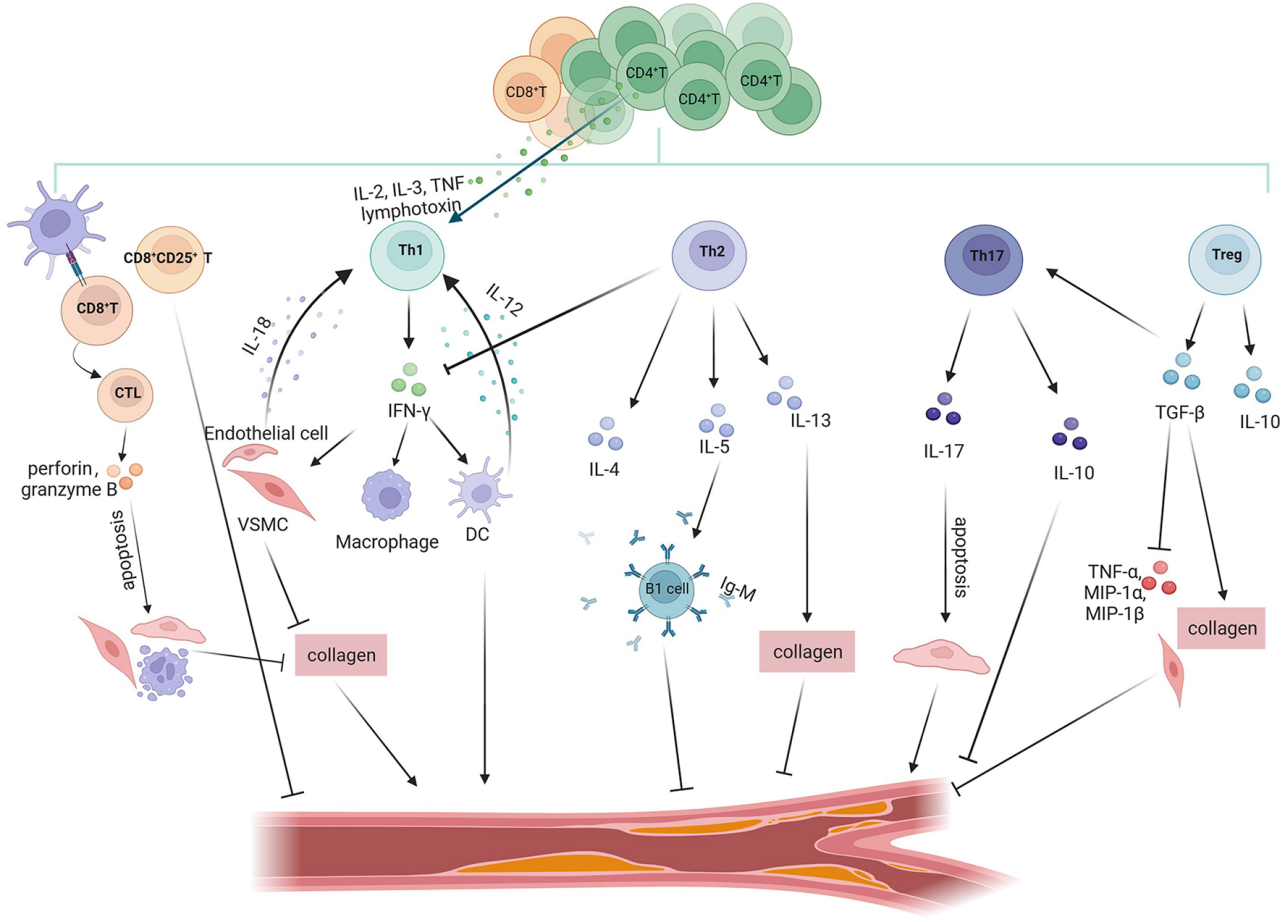
Role of T cell subsets in atherosclerosis. T cell subsets in the atherosclerotic process exert anti-atherosclerotic or pro-atherosclerotic effects by secreting different inflammatory factors. Pro-atherosclerosis: CD8^+^T cells recognise antigenic peptides presented by MHC-I to mature into CTL, which promote atherosclerosis through perforin/granzyme B-mediated apoptosis of VSMCs/ECs and monocytes and inhibition of collagen formation. Th1 cells secrete the signature cytokine INF-γ. IFN-γ stimulates VSMCs to impede collagen synthesis, which destabilises the plaque's thick fibrous cap and may lead to plaque rupture. INF-γ promotes the expression of inflammatory factors in DCs and macrophages. In addition, many CD4^+^T cells within atherosclerotic plaques express other pro-inflammatory cytokines associated with Th1 cells, such as IL-2, IL-3, TNF, and lymphotoxin, all of which activate Th1 cells, thereby accelerating inflammatory responses. IL-12 and IL-18 secreted by DCs and VSMCs/ECs exert proatherosclerotic effects by influencing the development and differentiation of Th1 cells. Anti-atherosclerotic: CD8^+^CD25^+^T inhibit the proliferation of CD4^+^T cells to exert antiatherosclerotic effects. Th2 cells produce the signature cytokines IL-4, IL-5 and IL-13. Among them, IL-5 promotes the development of IgM-secreting B1 cells; IL-13 has an atheroprotective effect through the increase in the collagen content of plaque components. Tregs produce the anti-inflammatory factors IL-10 and TGF-β to exert atheroprotective effects. TGF-β inhibits the expression of TNF-α, MIP-1*α* and MIP-1β, and promotes collagen formation in VSMCs to protect the stability of plaques. Controversial: Th17 cells express the signature cytokine IL-17, which exerts proatherosclerotic effects by promoting vascular endothelial cell senescence. However, TGF-β induces Th17 cells to produce IL-10 concomitantly with IL-17, which can be protective against atherosclerosis.

T cells, which are important parts of the adaptive immune response, are primarily separated into two groups: CD4^+^T cells and CD8^+^T cells. CD4^+^T cells can develop into specific Th cells (Th1, Th2, Th17) or Treg cells. Th cells and Treg cells have the ability to directly promote or inhibit inflammation in tissue-resident cells while also modulating the responses of other immune cells. Different CD4^+^T cell subsets have distinct transcriptional programs and cytokine-releasing patterns that can either exacerbate or mitigate AS. CD8^+^T cells participate in the adaptive immune response process and play an immunosurveillance role by mainly relying on the killing of target cells (Figure 3).

Th1 cells are the predominant subpopulation of Th cells in plaques, expressing the determinant transcription factor T-bet and secreting IFN-γ (63). The signature Th1 cell cytokine, IFN-γ, has been shown to have a pro-atherosclerotic effect on a number of cells in atherosclerotic lesions, including VSMCs, ECs, monocytes/macrophages, and DCs (64). For example, IFN-γ induces VSMCs to obstruct collagen production, which may lead to a rupture of the plaque by causing instability in the thick fibrous cap of the plaque. Mice that lack T-bet, its receptor, or IFN-γ are protected against AS (65). Compared to untreated mice, IFN-γ treatment in Apoe^−/−^ mice consistently promotes AS (66). Furthermore, a number of Th1-associated pro-inflammatory cytokines, including IL-2, IL-3, tumour necrosis factor (TNF), and lymphotoxin, are expressed by many CD4^+^T cells in atherosclerotic plaques in addition to IFN-γ. These cytokines have the ability to activate macrophages, T cells, and other cells, thereby quickening the inflammatory response (5). Apart from their ability to modulate Th1 cell function, cytokines crucial for Th1 cell differentiation and development, such as IL-12 and IL-18, also exhibit pro-atherosclerotic effects on this particular subgroup of T cells. Exogenous IL-12 injection led to an increase in atherosclerotic plaques in the aorta of mice (67). This discovery was consistent with the observation that anti-IL-12 antibody administration resulted in a 68% reduction in atherogenesis and an increase in collagen contents in atherosclerotic mice (68). IL-18-deficient Apoe^−/−^ mice had significantly smaller lesions, reduced local IFN-γ production, and decreased Th1 activity (69). However, direct injection of IL-18 recombinant protein promotes the development of AS in mice (70).

Th2 cells represent an additional subset of CD4^+^T cells that react to IL-4, IL-5, and IL-13 by starting the production of these cytokines in a positive feedback loop, while simultaneously suppressing IFN-γ expression. Studies have shown that IL-4 is associated with atherosclerotic inflammation resolution, and treatment with plaque-associated low concentrations of IL-4 enhanced the expression of inflammatory resolving factors (71); It has been discovered that IL-5 promotes the growth of B1 cells that can produce Ox-LDL-specific IgM to help clear lipids and lessen the formation of local foam cells (72), whereas IL-13 has been found to have an atheroprotective role by controlling macrophage activities for Ox-LDL clearance and raising the amount of collagen in plaque composition (73). The aforementioned investigations showed that Th2 cell-secreted IL-4, IL-5, and IL-13 are the main cytokines that exhibit atheroprotective properties in mice.

Regulatory T cells (Tregs) account for 5%–10% of the total number of CD4^+^T lymphocytes in the peripheral blood (74). Tregs play an atheroprotective role in modulating T cell-mediated immune responses by inhibiting T cell proliferation and secreting inhibitory cytokines, such as interleukin-10 (IL-10) and TGF-β (75). Adoptive cell therapy (ACT) using Tregs in Ldlr^−/−^ mice improves plaque stability by increasing collagen and VSMCs content and reducing plaque progression and lipid deposition (76). DEREG (regulatory T-cell depleted) mice, which carry a diphtheria toxin (DT) receptor controlled by the Foxp3 gene locus, were used on an AS-prone Ldlr^−/−^ background (77). Foxp3^+^ Tregs depletion exacerbates AS by increasing the proportion of pro-inflammatory IFN-γ-producing CD4^+^ effector T cells in the aorta. In addition to their direct functions within cells, Tregs' atheroprotective properties are also demonstrated by their characteristic cytokines. In a recent study, it was demonstrated that the progression of AS in human could be slowed by subcutaneous injection of IL-10/Treg-inducing small molecule-based formulation (78). Another cytokine that reduces inflammation is TGF-β. TGF-β, for instance, has been demonstrated to stabilize plaques by promoting collagen production in mice SMC (79). According to other research, overexpression of TGF-β inhibits the formation of plaque in mice aortic tissue by lowering the production of TNF-α and the T cell-attracting cytokines MIP-1*α* and MIP-1β (80). In conclusion, these data suggest that Tregs either at the cellular level or through the production of the anti-inflammatory cytokines IL-10 and TGF-β together exert a potent anti-inflammatory function and inhibit the development of atherosclerotic lesions. During atherosclerosis, some Tregs change into exTregs. By using scRNA-seq and CITE-seq analysis, it was discovered that the exTregs were cytotoxic and inflammatory CD4^+^ T cells (81). Functionally, human exTregs are cytotoxic and lack suppressive ability. They express inflammatory cytokines, cytotoxic effectors, chemokines (CCL3, CCL4, and CCL5), and chemokine receptors (including CCR5, CXCR2, CXCR3, CXCR4, and CX3CR1). When stimulated, exTregs increase the production of IFN-γ. Furthermore, it has been demonstrated that the size of atherosclerotic lesions is increased by the adoption of exTregs. Additionally, exTregs from CAD patients express more genes related to inflammation and cytotoxicity (81).

The preceding findings imply that among CD4^+^T cells, Th1 cells and exTregs have pro-atherosclerotic effects, and Tregs and Th2 cells have anti-atherosclerotic effects. However, the involvement of Th17 cells in AS is not as clear as that of Th1 cells, Tregs and Th2 cells. Th17 cells express the characteristic cytokine IL-17 (82). IL-17 stimulates the production of pro-inflammatory cytokines, including IL-6, granulocyte-colony stimulating factor, granulocyte-macrophage colony-stimulating factor, and chemokines, in immunological, endothelial, and stromal cells (82). These cytokines may also have pro-atherogenic effects. Furthermore, IL-17 has the ability to cause VECs senescence. The build-up of senescent VECs frequently results in oxidative stress and chronic inflammation, which causes endothelial dysfunction and accelerates the development of AS (83). It was demonstrated that overexpression of IL-17 significantly increases plaque vulnerability, characterized by the accumulation of lipids and T cells, along with a reduction in the number of VSMCs (84). Similarly, blocking IL-17-neutralizing antibodies attenuated plaque fragility, demonstrating the proatherosclerotic effect of IL-17 in mice (85). On the other hand, in a mice model, IL-6 and TGF-β stimulate a certain subset of Th17 cells that simultaneously generate IL-17 and IL-10, and IL-10 has atheroprotective properties (86).

Antigenic peptides presented by MHC-I are recognized by CD8^+^T cells. They can develop into cytotoxic T lymphocytes (CTL), which are capable of utilizing a variety of cytotoxic pathways to eliminate aberrant (such as cancerous) and virus-infected cells. Compared to healthy people, the blood of patients with coronary artery disease has higher concentrations of CD8^+^T cells that produce cytotoxins (87). CD8^+^T cells predominate in fibrous cap regions and outnumber CD4^+^T cells in advanced human atherosclerotic lesions (88). Apoe^−/−^ mice that had their CD8^+^T cells depleted by a particular antibody showed decreased macrophage aggregation in lesions and inhibited the development of atherosclerotic plaques (89). CD8^+^T lymphocytes promote the formation of vulnerable atherosclerotic plaques by causing apoptosis in VSMCs, ECs, and macrophages via perforin and granzyme B. This process results in the formation of necrotic cores and intensifies inflammation through the release of TNF-α. But in the atherosclerotic plaques of Apoe^−/−^ mice given a hypercholesterolemic diet, CD8^+^CD25^+^T cells were seen. The functional examination of splenic CD8^+^CD25^+^T cells from Apoe^−/−^ mice, by both flow cytometric analysis and CFSE-based proliferation experiments, demonstrated a suppressive behavior. Recipient mice with Apoe^−/−^ had much less AS after receiving an adoptive transfer of CD8^+^CD25^+^T cells, which also decreased the growth of splenic CD4^+^T cells (90). Consequently, developing innovative therapeutic strategies for AS may be made possible by comprehending the intricate function that CD8^+^T cells play in the disease.

Research from experimental models is the basis of our knowledge of T cell involvement in AS. It is difficult yet necessary to apply these discoveries to human disease. Immunotherapies that target T cells are attractive avenues for the development of new preventive and therapeutic approaches. Many studies are presently focused on creating Tregs-based treatments to modify the dysregulated immune response in AS, given the crucial role Tregs play in preserving immunological homeostasis *in vivo*. Enhancing Tregs *in vitro* for transmissible transfer is one potential method for using Tregs therapeutically. Animal studies have shown that *in vivo* injection of Tregs significantly reduces AS (91). However, due to a variety of reasons, there is currently not enough information available from human studies to evaluate the potential long-term consequences of transplanted Tregs. To guide clinical studies in atherosclerotic disease, more information from these ongoing trials is required. Tregs can be enhanced by *in vivo* induction in addition to *in vitro* amplification. Injecting immune complexes that target Tregs (IL-2/anti-IL-2) can increase Tregs *in vivo* (92). When Western-type diet-fed Ldlr^−/−^ mice were given the IL-2/anti-IL-2 combination, the number of IL-10-producing Tregs increased up to ten-fold in the circulation and various (lymphoid) organs. This increase in Tregs potently suppressed effector T cells and reduced the formation of early atherosclerotic lesions (93). The injection of antigens through a tolerogenic pathway is another often employed strategy to produce Tregs. It has been demonstrated that administering AS-relevant antigens, such as Ox-LDL (94), HSP60, and ApoB100 peptide (95), orally, nasally, or subcutaneously, suppresses AS in mice by boosting antigen-specific Tregs via the generation of tolerogenic DCs (96). In addition to the above immunotherapies targeting Tregs, it is possible that targeting Th1 cells may also be used to modulate the dysregulated immune response in AS. Therapeutic suppression of Th1 cells is a novel and promising approach to minimizing AS because of Th1's pro-atherosclerosis activity. But no clinically accessible treatment specifically targets Th1 cells (97). Th1 cell development from naïve T cells is effectively promoted by the cytokine IL-12. It has been shown that various IL-12 blocking antibodies are used in psoriasis treatment trials (98). More clinical studies are required to confirm if IL-12-specific antibodies may attenuate the atherosclerotic effects of Th1 cells in people and limit their differentiation. The role of the cell membrane-bound receptor known as the epidermal growth factor receptor (EGFR) in the development of lung cancer has been extensively studied. Recently, researchers have shown that Th1 cells in particular, which are leukocytes, also express EGFR (99). Treatment of Ldlr mice with EGFR inhibitors can reduce the development of AS and decrease T cell proliferation and activation (100) In order to tackle AS, EGFR may be another novel target to inhibit. To confirm if targeting Th1 cells can be used to treat AS in clinical practice, further trials must be conducted.

### B cells

The role of T cells in AS has been studied for decades, but the role of B cells in AS has been less well studied. Unlike T cells, only a small numbers of B cells can be found locally in AS and B cells play a key role in innate and adaptive immunity through their ability to produce antibodies and secrete cytokines. Major's team first discovered that B lymphocytes protect against AS (101). Contrary to earlier findings, new research has shown that inhibiting mature B cells efficiently inhibits T cell activation and slows the progression of AS (102).

**Figure 4:**
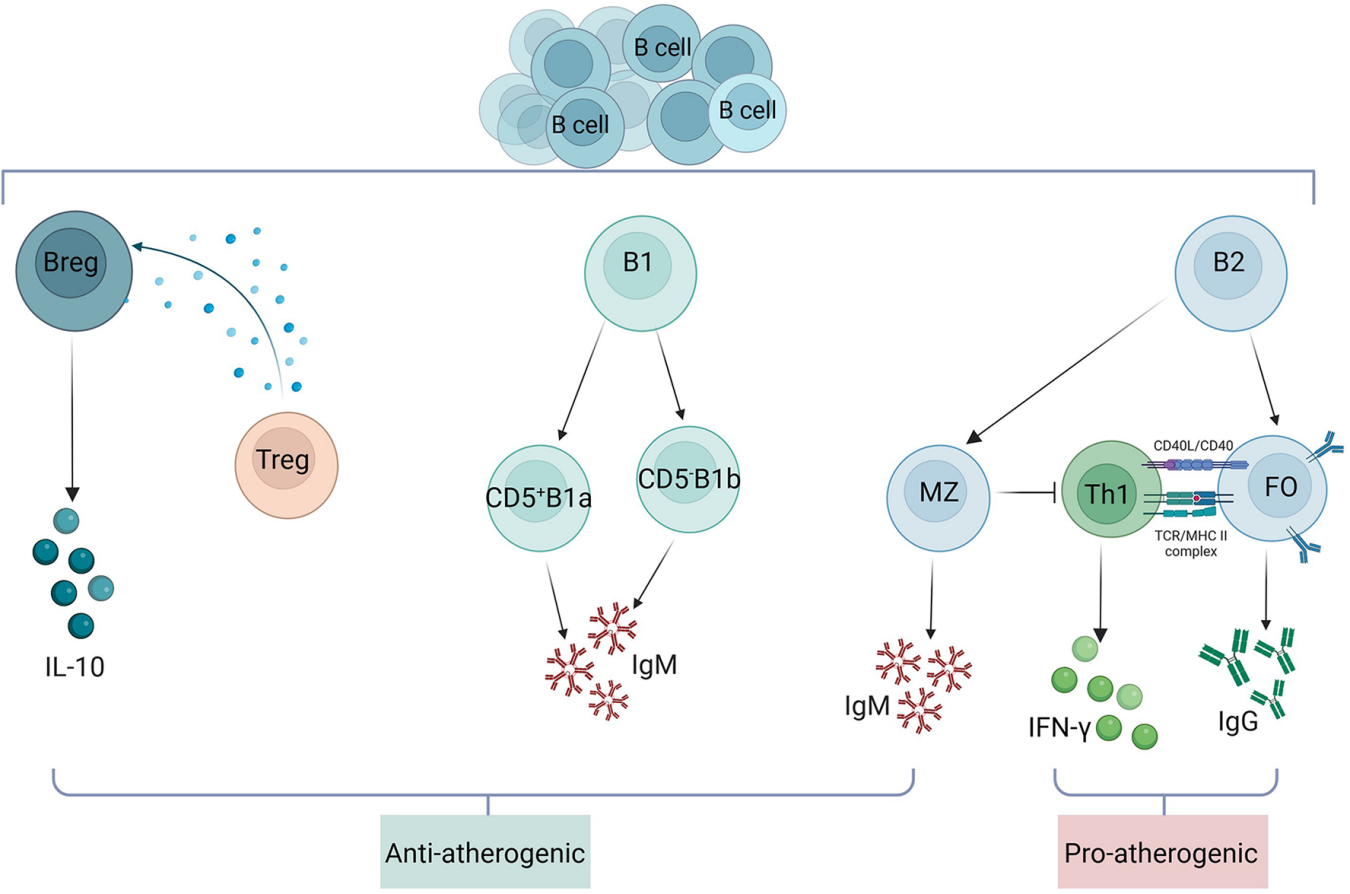
Role of B cell subsets in atherosclerosis. B cells within atherosclerotic plaques are divided into three main subsets: B1 cells, B2 cells and Breg cells. B1 cells are divided into CD5^+^B1a and CD5^−^B1b subpopulations by the surface marker CD5. Both B1a and B1b cells are atheroprotective by producing IgM. Breg cells exert immunosuppressive effects by secreting the cytokine IL-10. IL-10 production and Treg induction support the anti-atherosclerotic effect of Breg. B2 cells consist of FO and MZ B cells, and FO B cells exert their proatherosclerotic effects mainly through the production of IgG and activation of Th1 cells. MZ B cells exert atheroprotective effects by inhibiting Th cells and producing protective IgM.

As the understanding of B cells has advanced, studies have revealed that different B cell subsets have different functional roles in atherosclerotic disease (Figure 4). B cells within plaques are divided into three main subsets, B1 cells, B2 cells and Breg cells. B1 cells are divided into CD5^+^B1a and CD5^−^B1b subpopulations by the surface marker CD5. B1a and B1b cells have the ability to produce IgM antibodies that bind to OSE in LDL, apoptotic cells, or pathogen cell wall polysaccharides, hence exerting atheroprotective effects (103). Research has demonstrated that while a translocation of B1a or B1b cells decreases atherogenesis, a reduction in B1a cells exacerbates AS. Marginal Zone (MZ) B cells and Follicular (FO) B cells make up B2 cells. Th1 response and pro-inflammatory cytokine production are supported by FO B cells. Research has demonstrated that the primary mechanisms by which FO B lymphocytes promote AS are IgG production and Th1 cell activation (104). The smallest subset of B2 B cells, known as MZ B cells, prevent AS by suppressing Th cells and generating protective IgM antibodies (105). Breg cells exert immunosuppressive effects by secreting the cytokine IL-10. IL-10 production and Treg induction support the anti-atherosclerotic effects of Breg, and inhibition of IL-10 secretion promotes inflammatory cell infiltration and cytokine production and enhances AS in mice (106).

Clinical trials have employed B-cell targeted medicines to treat a range of illnesses, including systemic lupus erythematosus (SLE), rheumatoid arthritis (RA) and multiple sclerosis. B-cell therapy has potential atherosclerotic effects. Specific inhibition of the B cell surface receptor (BCR) signalling pathway reduces the release of pro-inflammatory chemokines, and therefore the use of BCR signalling inhibitors may be atheroprotective (107). However, to date, the cardiovascular effects of these drugs have mainly been an increased risk of arterial thrombosis (108). As a result, clinical trials of these agents are still limited and more studies and data are needed to validate them. In addition to targeting B-cell surface receptors, targeting B-cell survival-related receptors to regulate B-cell activity may also be a novel strategy for treating AS. BAFF and A proliferation-inducing ligand (APRIL) binding to cell surface receptors are two of the components that are essential for B-cell survival. When these ligands and antigens bind to the BCR, an intracellular signaling cascade is triggered, which increases B-cell survival and activates nuclear factor kB (109). It was discovered that BAFFR-deficient Ldlr^−/−^ mice had fewer B2 cells whereas IgM and B1a cell numbers were unchanged (110). Comparable results were seen in Apoe^−/−^ mice lacking BAFFR and Apoe^−/−^ mice given anti-BAFFR mAb treatment (111). These investigations demonstrated a reduction in the production of atherosclerotic lesions in mice, indicating the therapeutic benefit of targeting BAFF-BAFFR. Nevertheless, in hyperlipidemic mice, BAFF overexpression also enhanced the generation of anti-Ox-LDL IgM and markedly reduced AS (112). Therefore, further research is required to understand the B-cell subsets and processes that are impacted by medications that target BAFF. It has been shown that APRIL can confer atheroprotective effects on AS by binding to heparan sulfate proteoglycans to limit LDL retention, macrophage aggregation, and necrotic core formation (113). Therapeutic interventions aimed at BAFF/BAFFR and APRIL are primarily employed in the clinical context to address autoimmune diseases such as multiple sclerosis (MS), rheumatoid arthritis (RA), and systemic lupus erythematosus (SLE), as well as graft-vs.-host disease (114–116). Once the functions of APRIL and BAFF in AS are more clearly understood, these medications may be repurposed as therapies for AS.

## Therapeutic prospects

Statins are the traditional treatment for atherosclerosis, and with the development of new technologies and improvements in existing techniques, there are now more different therapeutic approaches applied to the treatment of atherosclerosis (Table 2). In clinical practice, the various statins primarily decrease cholesterol levels by distinct mechanisms of action. Hydroxy-methylglutaryl-CoA (HMG-CoA) reductase inhibitors are statins, first-line drugs that inhibit hepatic cholesterol synthesis (131). In addition to their lipid-lowering effects, statins have immunomodulatory effects. Statins shift the immune system in the vascular bed from Th1 cells to Th2 cells to exert anti-inflammatory effects (117). For patients with statin intolerance, ezetimibe is a non-statin drug that declines cholesterol by blocking its absorption (131). The combination of statins with ezetimibe reduced LPS-induced IL-6 and IL-1β production in monocytes of hypercholesterolemic patients (132). The pathophysiological significance of these effects on atherosclerosis needs to be further investigated. Although statins have beneficial immunomodulatory effects, they must be taken daily. Cardiovascular events are more likely to occur when combined with a high discontinuation rate. Thus, even aggressive statin therapy does not prevent many adverse events. Anti-atherosclerotic lipid modulation strategies are non-specific measures to inhibit immune cell function in plaques. Novel drug delivery systems now make it possible to specifically modify immune cell function.

**Table 2:** Different therapeutic approaches in AS.

Therapeutic approaches	Studies/clinical trials	Functions	Animal models
Statin therapy	HMG-CoA	Shift the immune system in the vascular bed from Th1 cells to Th2 cells	Human (117)
Stem cell therapy	AT-MSCs	Promote M2 polarization in human macrophages	Human (118)
	BM-MSCs	Inhibit activation and proliferation of Th1 and Th17 and promote differentiation of Tregs	Mouse (119)
Immunotherapy	Canakinumab	IL-1β antagonists inhibit inflammation	Human (120)
	Tocilizumab	IL-6 antagonists inhibit inflammation	Human (121)
	Infliximab	TNF-α antagonists inhibit inflammation	Human (122)
Exosome-based therapy	MSC-derived exosomes	Reduce macrophage infiltration in plaques; Promote macrophage M2 polarization	Mouse (123, 124)
	BMDM-derived exosomes	Inhibit inflammation; Promote macrophage M2 polarization	Mouse (125)
Nanoparticle (NP) -based therapy	TPCD NPs	Reduce internalization of Ox-LDL; Reduce foam cell formation in macrophages and VSMCs	Mouse (126)
	Mesoporous silica NPs loaded with the lipid-lowering drug	Inhibit inflammation	Mouse (127)
Natural product-based therapy	Quercetin	Inhibit inflammation	Mouse (128)
	Tanshinone IIA	Inhibit inflammation	Mouse (129)
	Lycopene	Inhibit inflammation	Mouse (130)

In recent years, nanoparticle (NP)-based therapy has shown great potential in AS. Nanomedicines can overcome the problem of rapid renal excretion and remain in the blood circulation for a longer period of time, thus achieving maximum therapeutic effect with minimum dose. The targeted delivery of nanomedicines to atherosclerotic plaques can be categorized into two main types (133). In one type of non-specific targeting, large gaps in dysfunctional ECs allow macromolecules and NPs to leak out of local areas of plaque neointima formation, leading to nanoparticle aggregation at the lesion. This allows nanodrugs to be targeted at different disease microenvironments. The cyclic polysaccharide β-cyclodextrin (abbreviated as TPCD) was covalently attached to the superoxide dismutase mimic Tempol and the removed hydroperoxide molecule of phenylboronic acid pinacol ester to create a broad-spectrum ROS scavenging material (126). It is easy to convert TPCD into NPs (TPCD NPs). After being injected intravenously, TPCD NPs passively targeted transport through defective endothelium and via inflammatory cells, accumulating in atherosclerotic lesions in Apoe^−/−^ mice. TPCD NPs decreased the internalization of Ox-LDL, which successfully reduced the development of foam cells in macrophages and VSMCs. Furthermore, by binding to certain cells or molecules at the lesion site through their surface ligands, NPs can achieve selective targeting, which enables the local therapeutic activity of nanomedicine to be poured into the plaque. An enzyme-responsive and macrophage-targeted drug delivery system has been studied to target the atherosclerotic microenvironment characterized by excessive inflammation and hyaluronidase (127). Mesoporous silica NPs were loaded with the lipid-lowering drug simvastatin and further linked to a hyaluronic acid coating, which gave the nanosystem hyaluronidase responsiveness and targeted to inflammatory macrophages. In addition, preliminary animal studies have shown that the established nanosystems have long plasma retention times and good biocompatibility *in vivo*, along with potent targeting, anti-foaming and anti-inflammatory effects. The application of targeted NPs in the management of AS has gained popularity. As nanotechnology advances and the demand for efficient treatments for cardiovascular disease grows, a wide range of novel nanomaterials along with various therapeutic agents and novel therapeutic targets, are being found and tested in animal models. Numerous of these have demonstrated targeted and efficient therapeutic effects, and a few have even been employed in clinical trials to treat AS (134, 135). Nano-interventions are an invasive treatment, and although the clinical treatment of NPs in terms of feasibility and safety has been initially demonstrated, there are still limitations in their stability, target specificity and toxicity in further clinical development.

Mesenchymal stem cells (MSCs) originated from different kinds of tissue are a group of cells possessing well-established self-renewal and multipotent differentiation properties as well as immunomodulatory and anti-inflammatory roles. Cardiac adipose tissue mesenchymal stromal cells (AT-MSCs) polarise human macrophages to an M2 anti-inflammatory phenotype, a function mediated in part by IL-6 (118). *in vitro*, studies have shown that bone marrow derived mesenchymal stem cells (BM-MSCs) suppress the activation and proliferation of Th1 and Th17 and promote the differentiation of Tregs (119). In addition to stem cell therapy, certain cell-derived exosomes may also play an immunomodulatory role. Immune cell-derived exosomes promote atherosclerosis, whereas exosomes derived from MSCs and bone marrow-derived macrophages (BMDM) attenuate atherosclerosis. Li et al. and Ma et al. reported that MSC-derived exosomes reduced the area of atherosclerotic plaques and macrophage infiltration in plaques, and promoted polarization of M2 macrophages in Apoe^−/−^ mice (123, 124). BMDM-derived exosomes contain miR-99a, miR-146b, and miR-378a, which have anti-inflammatory functions. These miRNAs regulate TNF-α and NF-*κ*B, which suppress inflammation and accelerate M2 polarization in BMDMs (125).

Several drugs are under investigation in pre-clinical or clinical trial: Canakinumab is IL-1β antagonists (120); Tocilizumab is IL-6 antagonist (121) and Infliximab is TNF-α antagonists (122). The above clinical drugs exert their anti-atherosclerotic effects mainly by inhibiting inflammatory factors. Several representative natural products (Quercetin (128), Tanshinone IIA (129), and Lycopene (130) exert their anti-atherosclerotic effects mainly through anti-inflammation and inhibition of LDL oxidation.

## Conclusions and perspectives

The formation, progression, and complications of AS are significantly influenced by immune cells, the cytokines they release, and the interactions between them. Moreover, in AS, studies have found different immune cells both on the side of lesion promotion and on the side of lesion inhibition. Different subtypes of the same immune cells each exert different pro- or anti-inflammatory effects, and the same immune cell subtypes sometimes exert different effects in different mouse treatments. This shows that the regulatory role of the immune machinery is very complex and that the action of a single type of cell, a single type of cytokine, is often influenced by other cells and cytokines. Thus, it is important to understand the role of a single immune cell. The utilization of single cell technologies holds promise for enhancing our basic comprehension of the intricate immunological systems that directly contribute to AS in humans.

With regard to AS, scRNA-seq has shown cellular heterogeneity. Examining the cellular makeup of health and illness led to the discovery of many disturbed-flow-induced cell types, including endothelial-to-mesenchymal transition (EndMT) cells and other disease-relevant cell types (136). These cells may be useful targets for prophylactic and therapeutic interventions. New cell subsets were identified by scRNA-seq, including TREM2^hi^ macrophages linked to fibrosis, catabolism, and lipid metabolism (51). Results from scRNA-seq further indicated that Tregs and M2-like macrophages promote the regressing process (97). Furthermore, the extraordinary plasticity of VSMCs and ECs was made evident by the combination of scRNA-seq with lineage tracing technologies. Disease-related stimuli cause VSMCs and ECs to produce distinct cell phenotypes, each of which has a distinct function in a range of illnesses (51). Other than the cells listed above, scRNA-seq also identified a few other cells during AS, including granulocytes, dendritic cells, and NK cells. However, the functions and phenotypes of these cells were not thoroughly examined, which may offer guidance for future AS-related scRNA-seq studies (11).

ScRNA-seq technology enables high-throughput and high-resolution analysis of individual cells in AS, revealing changes in the number, distribution, phenotype and function of different cell types in the vessel wall, as well as their interactions with each other. This data advances our knowledge of the pathophysiology of AS and helps identify novel targets and biomarkers for treatment. At the same time, scRNA-seq technology can be combined with other single-cell histological techniques to explore the pathological process of AS from multiple perspectives, reveal the mode of action of drugs, guide individualized treatment strategies, promote the development of precision medicine and provide new solutions for the targeted treatment of AS.
